# Dislocation of the Hip into the Thyroid Foramen

**Published:** 1860-01

**Authors:** 


2. Dislocation of the Hit into the Thyroid Foramen —Reduction by the Method of the Author.—On the 22d of December, 1859, we were called to a robust young man, upon whom part of a load of coal had fallen. The signs of dislocation into the Thyroid Foremer were very apparent, and the accident had occurred only an hour before. The reduction was effected in the following manner:

1. The patient was placed fully under the influence of chloroform. 2. A piece of wood, three and a half inches in diameter, surrounded by a pillow, was pressed against the perineum. 3. The members were used as levers, by seizing by the hands, at the ankles, and carrying them toward each other. During its reduction, the patient lay with his hips on the edge of the bed, the members extending over it. The knees were not flexed. The reduction was effected in ten seconds after force was applied, with ease.

This is the fourth case in which we have reduced this form of dislocation of the hip, by the hands alone. In none has any bad effect resulted. Three of these cases were noticed in the number of this Journal for Oct., 1859, but as the method is new, and the subject important, it is believed that each additional case will be of interest.

